# A Convenient Calibration Method for LRF-Camera Combination Systems Based on a Checkerboard

**DOI:** 10.3390/s19061315

**Published:** 2019-03-15

**Authors:** Zhuang Zhang, Rujin Zhao, Enhai Liu, Kun Yan, Yuebo Ma

**Affiliations:** 1 Institute of Optics and Electronics of Chinese Academy of Sciences, Chengdu 610209, China; zhangzhuang14@mails.ucas.ac.cn (Z.Z.); leh@ioe.ac.cn (E.L.); yankunioe@163.com (K.Y.); MYB_IOE@163.com (Y.M.); 2 University of Chinese Academy of Sciences, Beijing 100149, China

**Keywords:** LRF, camera calibration, extrinsic calibration, sensors combination

## Abstract

In this paper, a simple and easy high-precision calibration method is proposed for the LRF-camera combined measurement system which is widely used at present. This method can be applied not only to mainstream 2D and 3D LRF-cameras, but also to calibrate newly developed 1D LRF-camera combined systems. It only needs a calibration board to record at least three sets of data. First, the camera parameters and distortion coefficients are decoupled by the distortion center. Then, the spatial coordinates of laser spots are solved using line and plane constraints, and the estimation of LRF-camera extrinsic parameters is realized. In addition, we establish a cost function for optimizing the system. Finally, the calibration accuracy and characteristics of the method are analyzed through simulation experiments, and the validity of the method is verified through the calibration of a real system.

## 1. Introduction

In the field of measurements, a single sensor is seldom able to perform high-precision measurements by itself. Combined multi-sensor measurement schemes can effectively combine the characteristics of each sensor, leveraging the complementary advantages of sensors, and improving the accuracy and robustness of the measurement system. As [1] shows, laser range finders (LRFs) provide high-precision distance information, while camera can provide rich image information. The combination of LRFs and cameras has attracted wide attention, with interesting applications in navigation [2], human detection [3] and 3D texture reconstruction [4]. 

Compared with the current mainstream schemes combining scanning lasers and vision, the more challenging combination of 1-D laser ranging and vision has attracted the attention of researchers due to its low cost and wide applicability. The Shuttle Radar Topography Mission (SRTM) [5] realizes high-precision measurements of Interferometric Synthetic Aperture Radar (IFSAR) on long-range cooperative targets. Ordez [6] proposed a combination of camera and Laser Distance Meter (LDM) to estimate the length of a line segment in an unknown plane. Wu [7] applied this method to a visual odometry (VO) system and realized the application in a quasi-plane scene. In our previous work, we further extended this method and constructed a complete SLAM method based on laser-vision fusion [1].

Sensor calibration is the premise of data fusion, including the calibration of each sensor’s own parameters and the relationship of relative data between each sensor [8]. However, as a necessary prerequisite for high-precision measurements, the calibration technology of 1D laser-camera systems evolves seldom. The existing calibration algorithm based on scanning laser ranging has been unable to apply, but the traditional one-dimensional laser calibration algorithms require a high-precision manipulator laser interferometer and other complex equipment. 

In this paper, a simple and feasible high-precision laser and visual calibration algorithm is proposed, which can calibrate the parameters of laser and camera sensors through only simple data processing. Firstly, the camera parameters and distortion coefficients are determined using a non-iterative method. Then, the coordinates of the laser spot in the camera coordinate system are obtained by inversion of the laser image points in the image, and the initial values of external camera and laser ranging parameters are estimated. Finally, the parameters are optimized through the parameterization of the rotation matrix [9] and the Gröbner basis method [10]. Compared with the existing methods, the main contributions of this paper are as follows:

(1) The method proposed in this paper has wider applicability. It can be used for joint calibration of vision sensors and LRF from 1D to 3D.
(2) Compared with existing 1D laser-vision calibration methods, the proposed method can be realized using a simple chessboard lattice, without complicated customized targets and high-precision mechanical structures.
(3) The accuracy and usability of the proposed method are verified by simulation and observation experiments.

This paper is organized as follows: the existing methods related to our work are outlined in the following section. Section 3 and Section 4 describe the mathematical model and illustrate the proposed algorithm. In Section 5, we evaluate the solution of the simulation and observation experiments. Finally, conclusions and future are provided in Section 6. 

## 2. Related Work

For the extrinsic parameters between LRF and vision sensors, it is helpful to combine the high-precision distance information of laser ranging with the high lateral resolution of vision to achieve high-precision pose estimation. However, this method is mostly used to calibrate 2D or 3D LRFs and cameras. 

Vasconcelos [11] calibrated the camera-laser extrinsic parameters by moving a checkerboard freely. This method assumes that the internal parameters are known and accurate, and converts the external parameter calibration problem into a plane-coplanar alignment problem to reach an exact solution. Similar work includes Scaramuzza [12] and Ha [13]. Ranjith [14] realized the correlation and calibration of 3D LiDAR data and image data through feature point retrieval. Zhang [15] uses mobile LRF and visual camera to achieve self-calibration of their external parameters through motion constraints. Viejo [16] realized the correlation of the two sets of data by arranging control points, and calibrated the external parameters of 3D LiDAR and a monocular camera.

However, the above algorithms are mostly used to calibrate the external parameters of 2D or 3D scanning laser and vision systems, and cannot be used for 1D laser ranging without a scanning mechanism due to the lack of constraints. For the calibration of 1D LRF, the traditional method mostly realizes the correlation between the two by means of complex a manipulator or specific calibration target. For example, Zhu’s [17] calibration algorithm is used to calibrate the direction and position parameters of a laser range finder based on spherical fitting. The calibration accuracy is high, but the solution is highly customized and not universal. Lu [18] designed a multi-directional calibration block to calibrate the laser beam direction of a point laser probe on the platform of a coordinate measuring machine. Zhou [19] proposed a new calibration algorithm for serial coordinate measuring machines (CMMs) with cylindrical and conical surfaces as calibration objects. Similar calibration methods are used in the implementation of the LFR-camera slam method [1]. The relative rotation and translation of the two sensors’ coordinate systems are estimated through a high-precision laser tracker.

Although this method can achieve high accuracy, it requires the installation of sensors on precision measuring equipment, which has high calibration cost and complex operation, and cannot meet the needs of low-cost and fast landing scenarios such as existing robots. In 2010, Ordez [6] proposed a set method of cameras and LRF to measure short distances in the plane. In another study [20], the author introduces a preliminary calibration method for a digital camera and a laser rangefinder. The experiment involves the artificial adjustment of the projection center of the laser pointer, and only two laser projections are used. The accuracy and robustness of the calibration method are both problematic. After that, Wu et al. [7] proposed a two-part calibration method based on the Ransac scheme, and solved the corresponding linear equation in the image by creating the index table of laser spot. However, this method cannot be well applied to the case where the laser light is close to the optical axis of the camera, and the final accuracy evaluation criteria are not given.

Zhang [21] proposed a simple calibration method for camera intrinsic parameters, where the parameters were determined using a non-linear method, and high accuracy was achieved. Afterwards, based on Zhang’s framework, researchers improved accuracy and scene expansion by designing different forms of targets [22,23,24] and improving the calibration of the algorithm [25,26,27]. Hartly [28] introduced the distortion division model to correct the imaging distortion. On this basis, Hong [29] further explored the calibration method of large distortion cameras.

Currently, the calibration of omnidirectional cameras has attracted wide attention in order to improve the user’s degree of freedom and immersion in the virtual reality and autopilot. Li et al. [30] proposed a multi-phase camera calibration scheme based on random pattern calibration board. Their method supports the calibration of a camera system which comprise normal pinhole cameras. Gwon Hwan [31] proposed a new intrinsic calibration and extrinsic calibration method of omnidirectional cameras based on the Aruco marker and a Charuco board. The calibration structure and method can solve the problem of suing overly complicated procedures to accurately calibrate multiple cameras.

At the same time, the calibration board also plays an important role in the other calibration processs. Liu [32] studied different applications of lasers and cameras. The calibration method of multiple non-common-view cameras by scanning a laser rangefinder is proposed. In the literature, the correlation between laser distance information and camera images is established through a specific calibration plate, so as to realize the relative pose estimation between cameras. Inspired by Liu’s work [32], we establish a constraint of 1D laser and monocular vision by combining planar and coplanar constraints, so as to determine related external parameters. Considering that the camera imaging model has a direct impact on the calibration accuracy, we have improved Zhang‘s method [21] used in camera calibration by replacing the traditional polynomial model with the division distortion model, and solved the linear solution of the iterative optimization using variable least squares on the basis of Hartly [28] and Hong [29]. Thus, the problem of falling into local optimal solutions is avoided, and the calibration speed is greatly improved. Combining the above innovations, a convenient method for calibrating the parameters of the camera-laser measurement system is realized, which can complete the calibration of measurement systems, including camera internal parameters, distortion coefficients and camera-laser external parameters, in one operation.

## 3. Measurement Model

Previous researchers established relatively mature camera imaging and laser measurement models. We integrate the two mathematical models and construct a complete mathematical description of the coordinate system.

**Figure 1:**
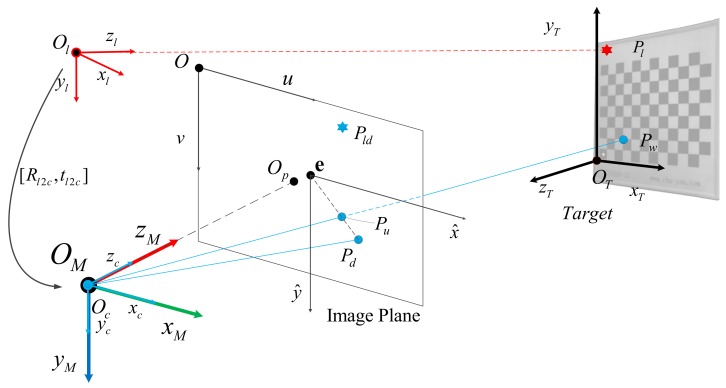
Measurement Model.

As shown in the Figure 1, $O_{C}$ is the camera coordinate system, $\vec{O_{C}Z_{C}}$ is the optical axis direction of the camera, O−uv is the image plane of the camera, $O_{T}$ is the coordinate system of the target itself, point $P_{l}$ is the spatial position of the laser spot, point $P_{w}$ is the spatial coordinate of the target control point and $O_{l}$ represents the coordinate system of 1D laser ranging. We set the camera coordinate system $O_{c}$ as the measurement coordinate system $O_{M}$ of the system. In the next part, we introduce the imaging model of the monocular camera and the 1D laser ranging model, and convert and fuse the data through extrinsic parameters $\left[\begin{array}{cc} R_{l2C} & T_{l2C} \end{array}\right]$.

### 3.1. Camera Imaging Model

In order to describe the imaging process of a monocular camera more accurately, we combine the lens distortion model with the aperture imaging model and introduce the shift of the distortion center e relative to the image center $O_{P}$ [28]. In the camera coordinate system, $O_{p}-xy$ is the physical coordinate system of the phase plane and O−uv represents the image coordinate system. Image center Op denotes the intersection of the optical axis and the image plane.

The ideal imaging process can be described as the process of transforming a point $P_{i}^{T}$ ($X_{i}^{T}={\left[\begin{array}{cccc} X_{i}^{T} & Y_{i}^{T} & Z_{i}^{T} & 1 \end{array}\right]}^{T}$) in the world coordinate system to the image plane imaging point $P_{i}^{u}$ ($x_{i}^{u}={\left[\begin{array}{ccc} u_{i}^{u} & v_{i}^{u} & 1 \end{array}\right]}^{T}$) through a projection relationship. The mathematical expression is as follows:

(1) $$\rho_{i}x_{i}^{u}=A_{C}T_{T2C}X_{i}^{T}=\left[\begin{array}{ccc} f_{u} & s & u_{0}\\ 0 & f_{v} & v_{0}\\ 0 & 0 & 1 \end{array}\right]\left[\begin{array}{cccc} r_{11} & r_{12} & r_{13} & t_{1}\\ r_{21} & r_{22} & r_{23} & t_{2}\\ r_{31} & r_{32} & r_{33} & t_{3} \end{array}\right]\left[\begin{array}{c} X_{i}^{T}\\ Y_{i}^{T}\\ Z_{i}^{T}\\ 1 \end{array}\right]$$

where $\rho_{i}$ is a named depth scale factor, the intrinsic matrix and $A_{C}$ is described by a five-parameter model; $f_{u},f_{v}$ are the focal lengths, ${\left[\begin{array}{cc} u_{0} & v_{0} \end{array}\right]}^{T}$ is the coordinate of the image center $O_{P}$, and s is the skew coefficient. $T_{T2C}$ is the transformation matrix relating $O_{T}$ to $O_{C}$ and it can be expressed as a rotation matrix $R_{T2C}$ combined with the translation vector $t_{T2C}$.

Due to lens design and processing, the actual imaging process is distorted. We introduce a division distortion model to improve our imaging. The mathematical expressions are as follows:

(2) $$x_{i}^{u}-e=\frac{x_{i}^{d}-e}{1+\lambda_{1}{\left[r_{i}^{d}\right]}^{2}+\lambda_{2}{\left[r_{i}^{d}\right]}^{4}+\ldots }$$

$x_{i}^{d}$ represents the actual position of projection point $P_{i}^{T}$, and its coordinates are $x_{i}^{d}={\left[\begin{array}{ccc} u_{i}^{d} & v_{i}^{d} & 1 \end{array}\right]}^{T}$; $\lambda_{1}$ and $\lambda_{2}$ are the distortion coefficients and $r_{d}$ represents the distance from point $x_{i}^{d}$ to the distortion center e, expressed as $r_{i}^{d}=\sqrt{{\left(u_{i}^{d}-du_{0}\right)}^{2}+{\left(v_{i}^{d}-dv_{0}\right)}^{2}}$.

In order to illustrate the method more clearly, the most important parameters used in this paper and their meaning are shown in Table 1.

### 3.2. LRF Model

The mathematical model of the 1D laser ranging module is relatively simple. The laser ranging module can output single point laser distance information by observing the reflected signal and calculating the optical path using image coherence [33]. The mathematical determination of the origin coordinate and laser direction of the laser ranging module allows the coordinate of the laser in the measurement coordinate system. In order to better represent the measurement results in the system measurement coordinate system $O_{M}$, we set up the European three-dimensional coordinate system $O_{l}$ for the LRF module. The laser emission direction is $\vec{O_{l}Z_{l}}$, the directions of $\vec{O_{l}X_{l}}$ are perpendicular and parallel to the $O_{C}-xy$ plane, and the directions of $\vec{O_{l}Y_{l}}$ are determined by the right-hand rule, as shown in Figure 1. The measured distance information $d_{i}^{l}$ represents the distance from the origin $O_{l}$ to the laser spot $P_{i}^{l}$.

In the process of extrinsic parameter calibration, the coordinate origin $O_{l}$ of the laser ranging coordinate system and the laser emission direction $\vec{O_{l}Z_{l}}$ need to be calculated. Finally, the conversion relations between camera measurement system $O_{M}$ and the LRF coordinate system $O_{l}$ are estimated, the rotation matrix $R_{l2M}$ and the translation vector $t_{l2M}$ are determined.

## 4. Methodology

Calibration of the measurement system is the process of determining the model parameters of the measurement system. For our system, through the measurement and imaging of a specific target, the model parameters of the measurement system are determined using the corresponding relationship between the coordinates of the control points and the image coordinates. The main parameters are the intrinsic parameters of the camera and the extrinsic parameters of between LRF and camera.

The specific calibration process is divided into three main steps: (1) the estimation of the camera distortion center e; (2) the intrinsic parameters $A_{C}$ and distortion coefficients $\lambda_{1}\lambda_{2}$ are decoupled and determined independently; (3) finding the extrinsic parameters $\left[\begin{array}{cc} R_{l2M} & t_{l2M} \end{array}\right]$ for translating the laser-vision coordinate system to the measurement coordinate system; (4) determining the optimal solution $\left(A_{C},\lambda_{1},\lambda_{2},\left[\begin{array}{cc} R_{l2M} & t_{l2M} \end{array}\right]\right)$ using the Gröbner basis method. In this section, we elaborate on the above.

### 4.1. The Center of Distortion

In many studies, it is usually assumed that the distortion center and the main point are in the same position, but Hartley [28] determined experimentally that there is a certain deviation between them. During the calibration process, we use a checkerboard as the calibration object, and extract the corners $P_{i}^{T}$ of the checkerboard as the control points for camera calibration. Since the corners are distributed on a plane, we set the $Z_{i}^{T}=0$ in the target coordinate system, in which case the imaging model can be expressed as:

(3) $$\rho_{i}x_{i}^{u}=PX_{i}^{T}=A[\begin{array}{cccc} r_{1} & r_{2} & r_{3} & t_{1} \end{array}]\left[\begin{array}{c} X_{i}^{T}\\ Y_{i}^{T}\\ 0\\ 1 \end{array}\right]$$

where $\left[\begin{array}{ccc} r_{1} & r_{2} & r_{3} \end{array}\right]$ is the column vector of rotation matrix $R_{T2C}$. The above equation can be simplified as:

(4) $$\rho_{i}x_{i}^{u}=HX_{i}^{T}=A_{C}[\begin{array}{ccc} \;r_{1} & r_{2} & t \end{array}]\left[\begin{array}{c} X_{i}^{T}\\ Y_{i}^{T}\\ 1 \end{array}\right]$$

Matrix H called the homography matrix, and expresses the mapping relation between the corner of the checkerboard and the image points. The coordinates of $P_{i}^{T}$ are abbreviated as $X_{i}^{T}={\left[\begin{array}{ccc} X_{i}^{T} & Y_{i}^{T} & 1 \end{array}\right]}^{T}$.

From the division model of Equation (2), we obtain:

(5) $$x_{i}^{d}=e+k_{i}\left(x_{i}^{u}-e\right),k_{i}=1+\lambda_{1}{\left[r_{i}^{d}\right]}^{2}+\lambda_{2}{\left[r_{i}^{d}\right]}^{4}+\ldots$$

We multiply the left side of the equations by ${\left[e\right]}_{\times }$ and combine it with Equation (4). In consideration of ${\left[e\right]}_{\times }e=0$:

(6) $${\left[e\right]}_{\times }x_{i}^{d}=k_{i}{\left[e\right]}_{\times }HX_{i}^{T},{\left[e\right]}_{\times }=\left[\begin{array}{ccc} 0 & -1 & dv_{0}\\ 1 & 0 & -du_{0}\\ -dv_{0} & du_{0} & 0 \end{array}\right]$$

We then multiply the left sides of the equations by ${\left[x_{i}^{d}\right]}^{T}$ and obtain:

(7) $${\left[x_{i}^{d}\right]}^{T}{\left[e\right]}_{\times }HX_{i}^{T}=0$$

Let $F_{H}={\left[e\right]}_{\times }H$. $F_{H}$ is called the fundamental matrix of distortion and is expressed as follows:

(8) $${\left[x_{i}^{d}\right]}^{T}F_{H}X_{i}^{T}=0,\;F_{H}=\left[\begin{array}{ccc} F_{11} & F_{12} & F_{13}\\ F_{21} & F_{22} & F_{23}\\ F_{31} & F_{32} & F_{33} \end{array}\right]$$

We can solve the values of the fundamental matrix $F_{H}$ using 8 pairs of corresponding corner points. The equation can be formulated as:

(9) $$Af_{H}=0$$

where:

(10) $$\begin{array}{l} A=\left[\begin{array}{ccccccccc} x_{1}^{d}X_{1}^{T} & x_{1}^{d}Y_{1}^{T} & x_{1}^{d} & y_{1}^{d}X_{1}^{T} & y_{1}^{d}Y_{1}^{T} & y_{1}^{d} & X_{1}^{T} & Y_{1}^{T} & 1\\ \vdots & \vdots & \vdots & \vdots & \vdots & \vdots & \vdots & \vdots & \vdots \\ x_{n}^{d}X_{n}^{T} & x_{n}^{d}Y_{n}^{T} & x_{n}^{d} & y_{n}^{d}X_{n}^{T} & y_{n}^{d}Y_{n}^{T} & y_{n}^{d} & X_{n}^{T} & Y_{n}^{T} & 1 \end{array}\right]\\ f_{H}=[\begin{array}{ccccccccc} F_{11} & F_{12} & F_{13} & F_{21} & F_{22} & F_{23} & F_{31} & F_{32} & F_{33} \end{array}] \end{array}$$

The corresponding equations are solvable using least square when the number of points is greater than 8 points. The corresponding distortion center e is the left null vector of $F_{H}$:

(11) $$e^{T}{\left[e\right]}_{\times }=0\Leftrightarrow e^{T}F_{H}=e^{T}{\left[e\right]}_{\times }H=0$$

So far, we have obtained the image coordinates of the distorted center e. The corresponding homography matrix H can be obtained using the fundamental matrix $F_{H}$.

### 4.2. Decoupling Camera Parameters

If the image coordinate origin $O_{P}$ is moved to the distortion center **e**, the new distortion center after translation is expressed as $\hat{e}={\left[\begin{array}{ccc} 0 & 0 & 1 \end{array}\right]}^{T}$. In the new coordinate system, Equation (8) is expressed as:

(12) $${\left[{\hat{x}}_{i}^{d}\right]}^{T}{\hat{F}}_{H}X_{i}^{T}=0$$

where ${\hat{x}}_{i}^{d}$ and ${\hat{F}}_{H}$ represent the transformed image coordinates $x_{i}^{d}$ and the fundamental matrix $F_{H}$. The transformation relationship is as follows:

(13) $${\hat{x}}_{i}^{d}=T_{e2\hat{e}}x_{i}^{d}\;,\;{\hat{F}}_{H}=T_{e2\hat{e}}F_{H},\;\mathrm{where}\;T_{e2\hat{e}}=\left[\begin{array}{ccc} 1 & 0 & -du_{0}\\ 0 & 1 & -dv_{0}\\ 0 & 0 & 1 \end{array}\right]$$

From the definition of ${\hat{F}}_{H}$:

(14) $${\hat{F}}_{H}={\left[e\right]}_{\times }\hat{H}=\left[\begin{array}{ccc} 0 & -1 & 0\\ 1 & 0 & 0\\ 0 & 0 & 0 \end{array}\right]\hat{H}$$

Let:

(15) $${\hat{F}}_{H}=\left[\begin{array}{c} {\hat{F}}_{1}\\ {\hat{F}}_{2}\\ {\hat{F}}_{3} \end{array}\right]\left[\begin{array}{ccc} {\hat{F}}_{11} & {\hat{F}}_{12} & {\hat{F}}_{13}\\ {\hat{F}}_{21} & {\hat{F}}_{22} & {\hat{F}}_{23}\\ {\hat{F}}_{31} & {\hat{F}}_{32} & {\hat{F}}_{33} \end{array}\right]$$

and:

(16) $$\hat{H}=\left[\begin{array}{c} {\hat{H}}_{1}\\ {\hat{H}}_{2}\\ {\hat{H}}_{3} \end{array}\right]\left[\begin{array}{ccc} {\hat{H}}_{11} & {\hat{H}}_{12} & {\hat{H}}_{13}\\ {\hat{F}}_{21} & {\hat{F}}_{22} & {\hat{H}}_{23}\\ {\hat{H}}_{31} & {\hat{H}}_{32} & {\hat{H}}_{33} \end{array}\right]$$

Equations (15) and (16) are then introduced into Equation (14):

(17) $${\hat{H}}_{1}={\hat{F}}_{2},{\hat{H}}_{2}=-{\hat{F}}_{1}$$

So far, the first two rows ${\hat{H}}_{1}\;{\hat{H}}_{2}$ of the homography matrix have been obtained. Referring to Equations (2) and (4), the image distortion after translation can be expressed as follows:

(18) $$\rho_{i}\frac{x_{i}^{d}}{1+\lambda_{1}{\left[r_{i}^{d}\right]}^{2}+\lambda_{2}{\left[r_{i}^{d}\right]}^{4}+\ldots }=HX_{i}^{T}$$

An equation set can be obtained after sorting out:

(19) $$\left[\begin{array}{c} {\hat{x}}_{i}^{d}{\left[X_{i}^{T}\right]}^{T}\left(-{\hat{F}}_{2}^{}X_{i}^{T}\right)\left[\begin{array}{ccc} {\left[{\hat{r}}_{i}^{d}\right]}^{2} & {\left[{\hat{r}}_{i}^{d}\right]}^{4} & \cdots \end{array}\right]\\ {\hat{y}}_{i}^{d}{\left[X_{i}^{T}\right]}^{T}\left({\hat{F}}_{1}^{}X_{i}^{T}\right)\left[\begin{array}{ccc} {\left[{\hat{r}}_{i}^{d}\right]}^{2} & {\left[{\hat{r}}_{i}^{d}\right]}^{4} & \cdots \end{array}\right] \end{array}\right]\left[\begin{array}{c} {\left[{\hat{H}}_{3}\right]}^{T}\\ \lambda_{1}\\ \lambda_{2}\\ \vdots \end{array}\right]=\left[\begin{array}{c} {\hat{F}}_{2}^{}X_{i}^{T}\\ -{\hat{F}}_{1}^{}X_{i}^{T} \end{array}\right]$$

**Figure 2:**
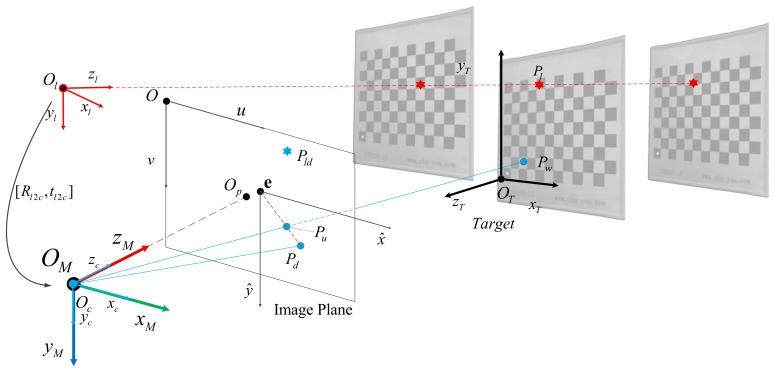
Changing of the azimuth and angle of the calibration plate and performing multiple measurements.

For Equation (19) and the combined Equation (17), two equations can be obtained for each pair of corner points. When the number of corresponding points N>=n+3 (where *n* denotes the number of distortion parameters), an overdetermined equation is obtained. This can be achieved by moving the target, as shown in Figure 2. The homography matrix $\hat{H}$ and the distortion coefficients $\lambda_{1}\lambda_{2}$ can be obtained by using the least square method.

### 4.3. Parameter Solution

From the perspective projection model, the imaging relationship of the translation sequence can be expressed as follows:

(20) $$\rho_{i}x_{i}^{u}=\rho_{i}{\left[T_{e2\hat{e}}\right]}^{-1}{\hat{x}}_{i}^{u}={\left[T_{e2\hat{e}}\right]}^{-1}\hat{H}X_{i}^{T}=HX_{i}^{T}$$

It is known that:

(21) $$H={\left[T_{e2\hat{e}}\right]}^{-1}\hat{H}=\left[\begin{array}{ccc} 1 & 0 & du_{0}\\ 0 & 1 & dv_{0}\\ 0 & 0 & 1 \end{array}\right]\hat{H}$$

Equation (19) can been solved to obtain $\hat{H}$. The initial homography matrix H can be calculated by substituting Equation (21). We set $\left[\begin{array}{ccc} \;r_{1} & r_{2} & t \end{array}\right]$$H=\left[\begin{array}{ccc} H_{1} & H_{2} & H_{3} \end{array}\right]=A_{C}[\begin{array}{ccc} \;r_{1} & r_{2} & t \end{array}]$. By using the orthogonality and normality of rotation matrix $\left[\begin{array}{ccc} \;r_{1} & r_{2} & r_{3} \end{array}\right]$, we obtain:

(22) $$\left\{\begin{array}{c} r_{1}r_{2}=0\\ r_{1}r_{1}=r_{2}r_{2} \end{array}\right.\Leftrightarrow \left\{\begin{array}{c} {\left[H_{1}\right]}^{T}{\left[A_{C}\right]}^{-T}{\left[A_{C}\right]}^{-1}H_{2}=0\\ {\left[H_{1}\right]}^{T}{\left[A_{C}\right]}^{-T}{\left[A_{C}\right]}^{-1}H_{1}={\left[H_{2}\right]}^{T}{\left[A_{C}\right]}^{-T}{\left[A_{C}\right]}^{-1}H_{2} \end{array}\right.$$

Therefore, three images are needed to find five unknowns in the camera intrinsic parameter matrix $A_{C}$. If the camera collects n images from different directions for calibration, a set of linear equations containing 2n constrained equations can be established, which can be written in matrix form as follows:

(23) Vb=0

where V is the coefficient matrix and b is the variable to be solved, with:

(24) $$b=[\begin{array}{cccccc} B_{11} & B_{12} & B_{13} & B_{22} & B_{23} & B_{33} \end{array}]$$

(25) $$B={\left[A_{C}\right]}^{-T}{\left[A_{C}\right]}^{-1}=\left(\begin{array}{ccc} B_{11} & B_{12} & B_{13}\\ B_{21} & B_{22} & B_{23}\\ B_{31} & B_{32} & B_{33} \end{array}\right)$$

The solvable camera intrinsic parameters are:

(26) $$\begin{array}{l} v_{0}=\left(B_{12}B_{13}-B_{11}B_{23}\right)/\left(B_{11}B_{22}-B_{12}^{2}\right)\\ \lambda =B_{33}-\left[B_{13}^{2}+v_{0}(B_{12}B_{13}-B_{11}B_{23})\right]/B_{11}\\ f_{u}=\sqrt{\lambda /B_{11}}\\ f_{v}=\sqrt{\lambda B_{11}/\left(B_{11}B_{22}-B_{12}^{2}\right)}\\ s=-B_{12}f_{u}^{2}f_{v}/\lambda \\ u_{0}=kv_{0}/f_{v}-B_{13}f_{u}^{2}/\lambda \end{array}$$

Similarly, the camera parameters can be obtained:

(27) $$[\begin{array}{cccc} r_{1} & r_{2} & r_{3} & t_{1} \end{array}]=\left[\begin{array}{cccc} \frac{{\left[A_{C}\right]}^{-1}H_{1}}{\left|{\left[A_{C}\right]}^{-1}H_{1}\right|} & \frac{{\left[A_{C}\right]}^{-1}H_{2}}{\left|{\left[A_{C}\right]}^{-1}H_{3}\right|} & r_{1}\times r_{2} & \frac{{\left[A_{C}\right]}^{-1}H_{3}}{\left|{\left[A_{C}\right]}^{-1}H_{3}\right|} \end{array}\right]$$

In the case of obtaining the parameters outside the target, the spatial coordinate $X_{l}^{C}={\left[\begin{array}{cccc} X_{l}^{C} & Y_{l}^{C} & Z_{l}^{C} & 1 \end{array}\right]}^{T}$ of the laser spot $P_{l}$ in the camera coordinate system can be found by solving the known plane equation $\mathbb{Q}(X^{C},Y^{C},Z^{C})$ in the direction obtained by connecting the ray and the target from the camera optical center $O_{C}$ to the ideal image point coordinate $x_{l}^{u}={\left[\begin{array}{ccc} u_{l}^{u} & v_{l}^{u} & 1 \end{array}\right]}^{T}$. Moving the calibration board along the laser direction, the spatial position of laser spot can be obtained at different distances after multiple acquisitions. By processing the data, the laser beam can be straight in the camera coordinate system.

Through data processing, the linear equation $\mathbb{C}(X^{C},Y^{C},Z^{C})$ of the laser beam in the camera coordinate system can be obtained in the form of Equation (29). By combining the distance information DL obtained through laser ranging, the spatial coordinates of the laser origin in the camera coordinate system can be obtained, and the transformation relationship $\left[\begin{array}{cc} R_{l2M} & t_{l2M} \end{array}\right]$ between the laser system and camera system can be estimated:

(28) $$\mathbb{Q}(X^{C},Y^{C},Z^{C}):\left[\begin{array}{cccc} A_{Q} & B_{Q} & C_{Q} & D_{Q} \end{array}\right]{\left[\begin{array}{cccc} X^{C} & Y^{C} & Z^{C} & 1 \end{array}\right]}^{T}=0$$

(29) $$\mathbb{C}(X^{C},Y^{C},Z^{C}):\frac{X_{li}^{C}-X_{l0}^{C}}{A_{l}}=\frac{Y_{li}^{C}-Y_{l0}^{C}}{B_{l}}=\frac{Z_{li}^{C}-Z_{l0}^{C}}{C_{l}}$$

where $X_{l0}^{C}=\frac{1}{n}\sum X_{li}^{C}$, $Y_{l0}^{C}=\frac{1}{n}\sum Y_{li}^{C}$, $Z_{l0}^{C}=\frac{1}{n}\sum Z_{li}^{C}$. Combining with Equation (27), we have:

(30) $$\mathbb{Q}(\begin{array}{cc} R_{T2M}, & t_{T2M} \end{array})=\left[\begin{array}{cccc} A_{Q} & B_{Q} & C_{Q} & D_{Q} \end{array}\right][\begin{array}{cccc} r_{1} & r_{2} & r_{3} & t_{1} \end{array}]{\left[\begin{array}{cccc} X^{T} & Y^{T} & Z^{T} & 1 \end{array}\right]}^{T}$$

By combining with the imaging model, the linear equation between laser spot and camera light center can be expressed in two-point form:

(31) $$\frac{X^{C}}{u_{l}^{u}}=\frac{Y^{C}}{v_{l}^{u}}=\frac{2\cdot Z^{C}}{f_{u}+f_{v}}$$

Equations (30) and (31) are solved simultaneously, the only solution of which $X_{l}^{C}={\left[\begin{array}{cccc} X_{l}^{C} & Y_{l}^{C} & Z_{l}^{C} & 1 \end{array}\right]}^{T}$ is the coordinate of the laser spot on the target in the camera coordinate system.

After many measurements, the linear equation can be expressed as a series of spatial point sets $\left\{\left.X_{li}^{C}\right|\;X_{li}^{C}={\left[\begin{array}{cccc} X_{li}^{C} & Y_{li}^{C} & Z_{li}^{C} & 1 \end{array}\right]}^{T},i=1,2,3,\cdots \right\}$, as shown in Figure 2. Constraints can be applied using a point-line relationship to solve the linear equation $\mathbb{C}(X^{C},Y^{C},Z^{C})$ corresponding to laser rays, such as:

(32) $$\left[\begin{array}{ccc} Y_{li}^{C}-Y_{l0}^{C} & -\left(X_{li}^{C}-X_{l0}^{C}\right) & 0\\ 0 & -\left(Z_{li}^{C}-Z_{l0}^{C}\right) & Y_{li}^{C}-Y_{l0}^{C} \end{array}\right]\left[\begin{array}{cc} A_{l} & 0\\ B_{l} & B_{l}\\ 0 & C_{l} \end{array}\right]=0$$

A space point can provide two constraints, and we need at least two space points to solve the equation and estimate the linear equation $\mathbb{C}(X^{C},Y^{C},Z^{C})$. Finally, the laser origin position is determined on the line by calculating the distance information obtained by ranging according to the coordinate system established before and using the relative transformation matrix of laser-camera $\left[\begin{array}{cc} R_{T2M} & t_{T2M} \end{array}\right]$.

### 4.4. Optimization of Solution

The above process does not involve any iteration. The camera internal parameters and laser-camera external parameters can be found using least squares. The calculation speed is fast and local minima can be effectively avoided effectively. If we want to obtain higher accuracy, we can take the calculated value as the initial value, and further improve the calibration accuracy of the system through the non-linear optimization method.

Given n calibrated images, each image has m corners $x_{i}^{d}$ and one laser projection point $x_{l}^{d}$. The following objective functions are then constructed:

(33) $$E(A_{C},\lambda_{1},\lambda_{2},R_{T2M},t_{T2M})=\sum_{i=1}^{n}\left(\sum_{j=1}^{m}\left|x_{i,j}^{d}-Pro(X_{j}^{T})\right|+\gamma \left|x_{l,j}^{d}-Pro(d_{i}^{l})\right|\right)$$

where $Pro(X_{j}^{T})$ and $Pro(d_{i}^{l})$ represent the projection functions of corner points $X_{j}^{T}$ and laser spot $X_{l}^{C}$ under the division distortion model, and γ is a named weight coefficient that denotes the contribution of corner and laser points to errors, generally speaking γ=5.

Using Cayley-Gibbs-Rodriguez (CGR) [9] to parameterize the rotation matrix R, the latter can be expressed as a function of the CGR parameters $s=\left[\begin{array}{ccc} s_{1} & s_{2} & s_{3} \end{array}\right]$:

(34) $$R=\frac{1}{1+s_{1}{}^{2}+s_{2}{}^{2}+s_{3}{}^{2}}\left[\begin{array}{ccc} 1+s_{1}{}^{2}-s_{2}{}^{2}-s_{3}{}^{2} & 2s_{1}s_{2}-2s_{3} & 2s_{1}s_{3}+2s_{2}\\ 2s_{1}s_{2}+2s_{3} & 1-s_{1}{}^{2}+s_{2}{}^{2}-s_{3}{}^{2} & 2s_{2}s_{3}-2s_{1}\\ 2s_{1}s_{3}-2s_{2} & 2s_{2}s_{3}+2s_{1} & 1-s_{1}{}^{2}-s_{2}{}^{2}+s_{3}{}^{2} \end{array}\right]$$

The problem is then transformed into an unconstrained optimization problem. The automatic Gröbner basis method [10] is used to solve Equation (32), and the minimum solution $E_{\min}({\tilde{A}}_{C},{\tilde{\lambda }}_{1},{\tilde{\lambda }}_{2},{\tilde{R}}_{l2M},{\tilde{t}}_{l2M})$ can be obtained. A nonlinear optimization method is used to further improve the accuracy and stability of the solution.

In this part, we have completed the estimation of the optimal solution of all parameters, including the camera intrinsic parameter matrix ${\tilde{A}}_{C}$, distortion coefficient ${\tilde{\lambda }}_{1}{\tilde{\lambda }}_{2}$ and laser-camera external parameters $\left[\begin{array}{cc} {\tilde{R}}_{l2M} & {\tilde{t}}_{l2M} \end{array}\right]$.

## 5. Experiment and Analysis

In this part, we evaluate the calibration methods of the camera internal parameters and camera-laser external parameters. The effectiveness and influencing factors of the proposed system calibration algorithm are analyzed through computer simulation experiments, while the measurement system is calibrated through observation experiments. In order to better evaluate the calibration results, we refer to the re-projection error [34] evaluation method in the camera calibration process, and unify the laser spot and target corner to establish the following error evaluation function:

(35) $$E_{dr}=\frac{1}{m\cdot n}\sum_{i=1}^{n}\left(\sum_{j=1}^{m}\left|x_{i,j}^{d}-Pro(X_{j}^{T})\right|+\gamma \left|x_{l,i}^{d}-Pro(d_{i}^{l})\right|\right)$$

The re-projection error *E_dr_* is an important metric of the calibration results: the smaller *E_dr_* is, the better the calibration results are.

### 5.1. Simulation Result

For the simulation experiment, we used the MATLAB R2016a software for Windows 10. The relevant parameters of the simulation system are shown in Table 2. In the measurement system, the laser direction is parallel to the optical axis of the camera and a 50 mm offset in the $\vec{O_{M}X_{M}}$ direction is arranged. 

**Figure 3:**
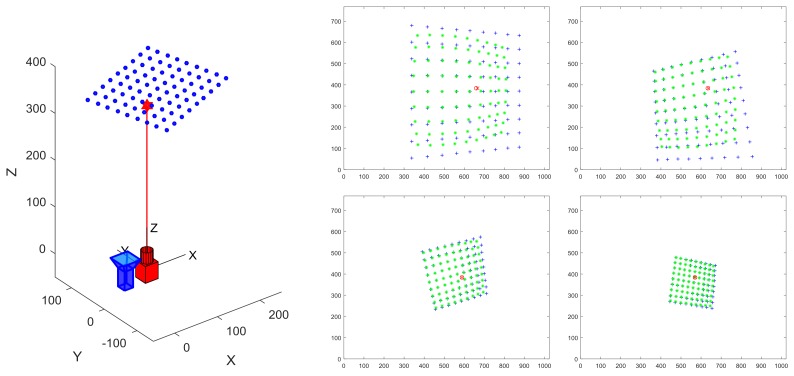
The imaging illustration. (**Left**) Schematic diagram of a simulated scenario. (**Right**) The generated image. The blue dots represent the ideal image points, the green dots represent the added distortion points, the red X represents the ideal image point of laser and the red circle is the distorted laser projection point.

The target is shown in the Figure 3, where the blue dots represent the corners of the checkerboard lattice, evenly distributed in the plane, and the adjacent corners are 15 mm apart. The relative position between the target and the system is randomly generated by the system within a given range.

Throughout the experiment, we compare the estimated values from each calculation with the real values set by simulation, and evaluate the accuracy of the algorithm by calculating the deviation between the two. The error is expressed as follows:

(36) $$\left\{\begin{array}{l} E_{fu}=\frac{\left|{\tilde{f}}_{u}-f_{u}\right|}{f_{u}},E_{fv}=\frac{\left|{\tilde{f}}_{v}-f_{v}\right|}{f_{v}}\\ E_{u0}=\frac{\left|{\tilde{u}}_{0}-u_{0}\right|}{u_{0}},E_{u0}=\frac{\left|{\tilde{v}}_{0}-v_{0}\right|}{v_{0}}\\ E_{R}=\max_{i=1}^{3}\left|\arccos\left({\tilde{r}}_{i}\cdot r_{i}\right)\right|\\ E_{t}=\left|{\tilde{t}}_{l2M}-t_{l2M}\right| \end{array}\right.$$

Kopparapu et al., confirmed [32] that noise has a significant impact on calibration accuracy. We add $\omega_{noise}∼Gauss\left(0,\Sigma_{noise}\right)$ Gaussian noise to the simulated projection image, where $\Sigma_{noise}$ is the standard deviation of the Gaussian distribution. In the simulation, the standard deviation $\Sigma_{noise}$ of noise increases gradually in the range of 0.1 to 1.5 pixel. For each $\omega_{noise}$ X distribution, we performed 100 independent experiments, and obtained the average value of calibration error as the statistical result.

**Figure 4:**
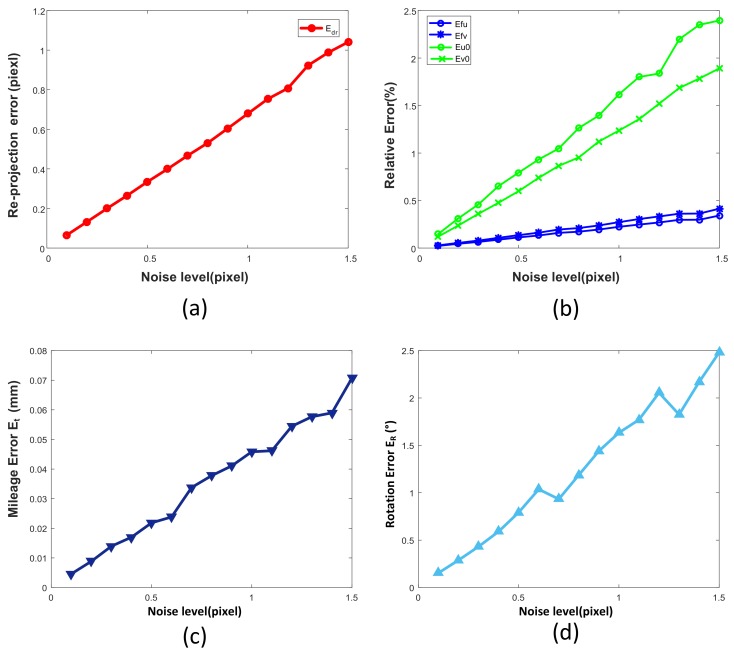
Simulation results for different image noise levels. (**a**) The re-projection error $E_{dr}$; (**b**) The effects of noise on intrinsic parameters such as $E_{fu}$, $E_{fv}$, $E_{u0}$, $E_{u0}$; (**c**) mileage error $E_{t}$ for different noise levels; (**d**) effects of noise on rotation error $E_{R}$.

The results are shown in Figure 4. It can be seen that with the increase of noise, the deviation between the calibration parameters and the true value increases linearly. When the corner extraction noise is 0.5 pixels, the system calibration error is about 0.2, the focal length deviation is 0.1%, and the main point deviation is about 0.8%. In terms of extrinsic parameters, the translation error also follows a linear distribution, but the fluctuation is more obvious. It can be seen that the system is sensitive to the internal parameters. At the same time, under the corner extraction error of 0.5 pixels, the translation error is about 1 mm and the rotation error is 0.02 degrees.

**Figure 5:**
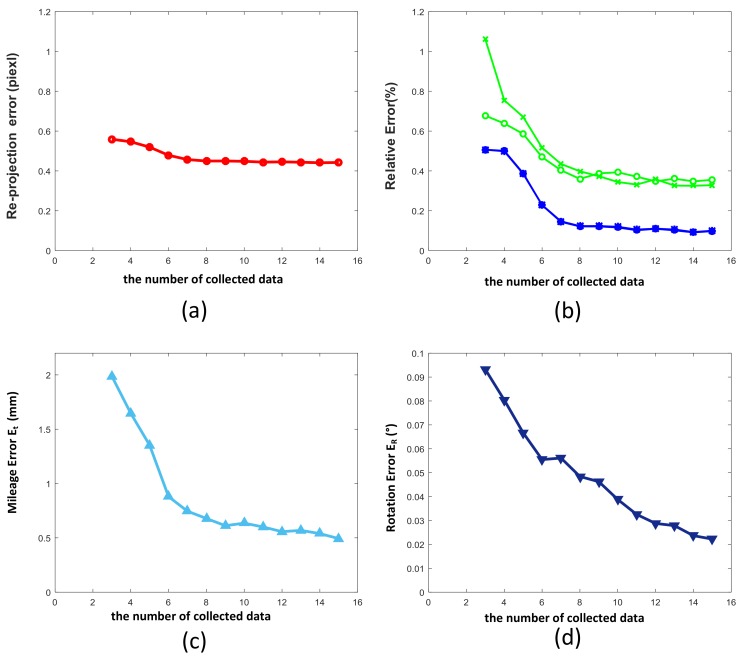
The simulation results for different numbers of collected data. (**a**) Re-projection error $E_{dr}$; (**b**) Effects of the number of data on intrinsic parameters such as $E_{fu}$, $E_{fv}$, $E_{u0}$, $E_{u0}$; (**c**) Mileage error $E_{t}$ for different noise levels; (**d**) Effect of the number of data on rotation error $E_{R}$.

In addition, we analyzed the impact of the number of collected data on the calibration accuracy, and set the calibration data to gradually increase from the minimum of three groups of image distance data to 15 groups. The results are shown in the Figure 5. With the increase of calibration data, the re-projection error remains almost stable, but the accuracy of the estimated system variables is significantly improved. When the number of data increases to 8, the decline of the correlation error slows down. Therefore, sufficient calibration data collected in a certain range can help to improve the accuracy of system calibration. However, after reaching a certain number, the effect gradually decreases, and so 8–10 groups of data are appropriate.

**Figure 6:**
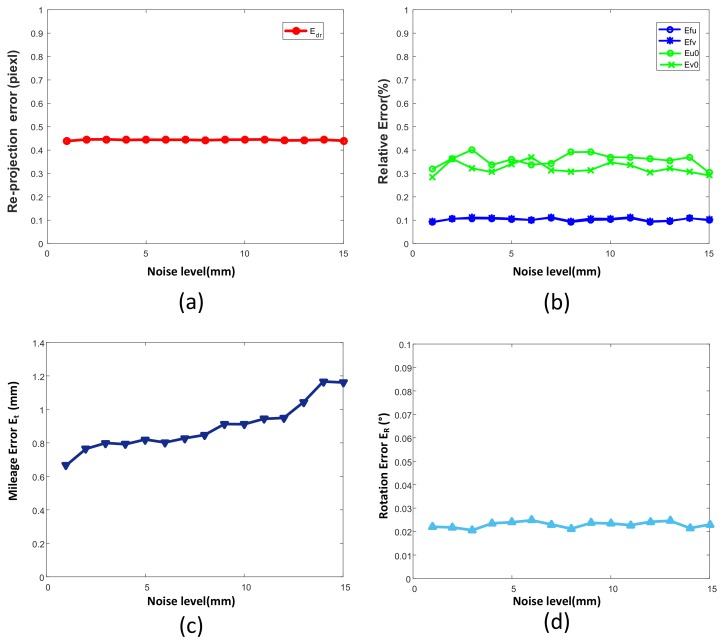
Simulation results of different distance noise levels. (**a**) Re-projection error $E_{dr}$; (**b**) Effects of noise on intrinsic parameters such as $E_{fu}$, $E_{fv}$, $E_{u0}$, $E_{u0}$; (**c**) Mileage error $E_{t}$ with different noise; (**d**) the effects of noise on rotation error $E_{R}$.

We also analyzed the influence of the measurement error of the laser ranging system on the calibration accuracy. The Gaussian-distributed noise $\omega_{d}∼Gauss\left(0,\Sigma_{d}\right)$ was added to the ranging error, and the standard deviation $\Sigma_{d}$ was changed gradually from 1 mm to 15 mm. We calculated the calibration errors of the parameters of the system at each noise level. As shown in Figure 6, except for the linear relationship between translation vector and distance error, the other parameters hardly change with the increase of error.

### 5.2. Real Experiment

**Figure 7:**
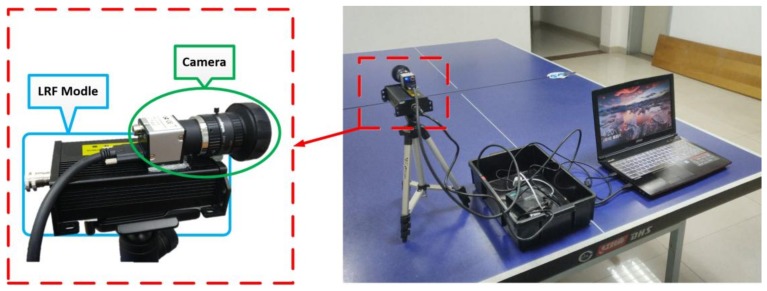
Measurement system combining 1D LRF and camera.

In the actual experiment, we built a measurement system with a 1D laser-camera combination, and calibrated the system with the method proposed in this paper. As shown in Figure 7, the system is composed of a MER-131-210U3C camera and a SKD-100 laser ranging system. The related parameters are shown in Table 3.

**Figure 8:**
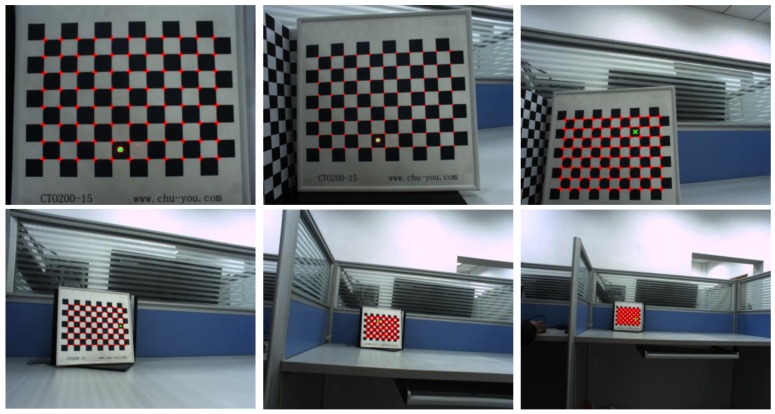
Samples of images used for the real experiment.

The system was calibrated using a calibration board composed of 11×8 square chessboard lattices with a distance of 15 mm between corners. The iterative Harris algorithm was used to extract the checkerboard corner coordinates (red +) from the calibrated image accurately, and the centroid method was used to extract the image coordinates (green ×) of the laser spot. The accuracy can reach sub-pixel level. The results of 12 images collected at different distances from 150 mm to 1500 mm are shown in Figure 8.

In order to verify the accuracy of our calibration method, we compared the internal parameters obtained with the classical Zhang [21] calibration method and the Li’s method [30]. In the calibration process of Bo‘s method, we replaced the original random corner matching process by directly inputting the coordinates of checkerboard lattices into the program, but still retain the complete algorithm for camera parameter determination. The results are shown in Table 4, where the accuracy of the intrinsic parameters obtained by the calibration methods are compared. The calibration accuracy is evaluated using the re-projection error [34] and expressed as:

(37) $$E_{rp}=\frac{1}{m}\sum_{j=1}^{m}\sqrt{{\left|x_{j}^{d}-Pro(X_{j}^{T})\right|}^{2}}$$

**Figure 9:**
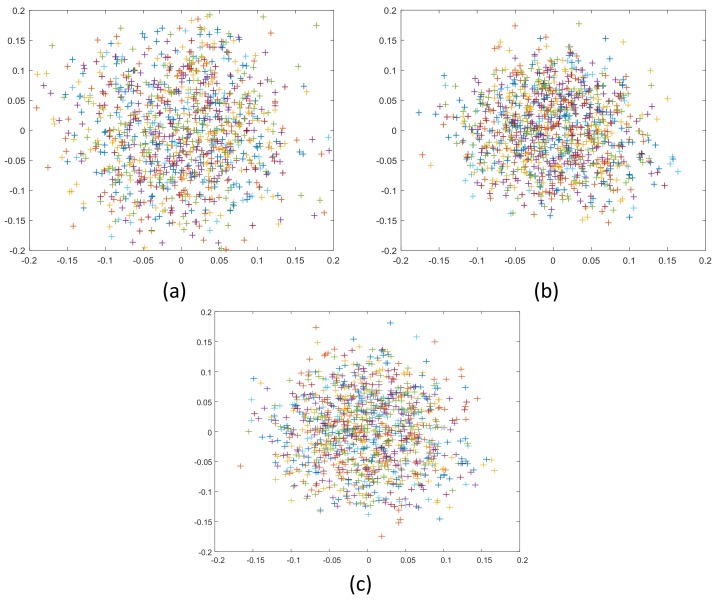
Re-projection error distribution for different images marked as different colors: (**a**) Zhang’s method [21]; (**b**) proposed method; (**c**) Li’s method [30].

From the calibration results in Figure 9 and Table 4, we see that our method and Zhang’s method [21] have similar calibration results in camera intrinsic parameters. Because the distortion models used by the two methods are different, the physical meanings of the distortion coefficients are different, so it is meaningless to compare them. Judging from the re-projection error, our method is slightly better than Zhang’s calibration algorithm. This proves the effectiveness of our calibration algorithm.

**Figure 10:**
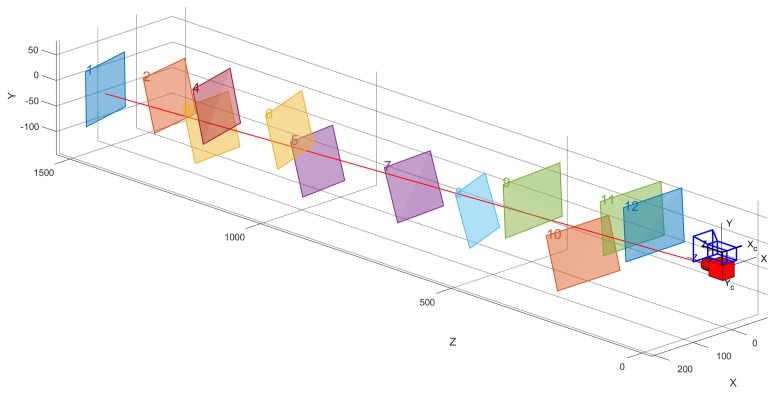
Visualization of extrinsic parameters $\left[\begin{array}{cc} {\tilde{R}}_{l2M} & {\tilde{t}}_{l2M} \end{array}\right]$.

At the same time, the extrinsic parameters $\left[\begin{array}{cc} {\tilde{R}}_{l2M} & {\tilde{t}}_{l2M} \end{array}\right]$ of the laser-camera combination of the measurement system are also calculated and the calibration results were evaluated using the evaluation function set Equation (37). The results are shown in Table 5 and Figure 10.

**Figure 11:**
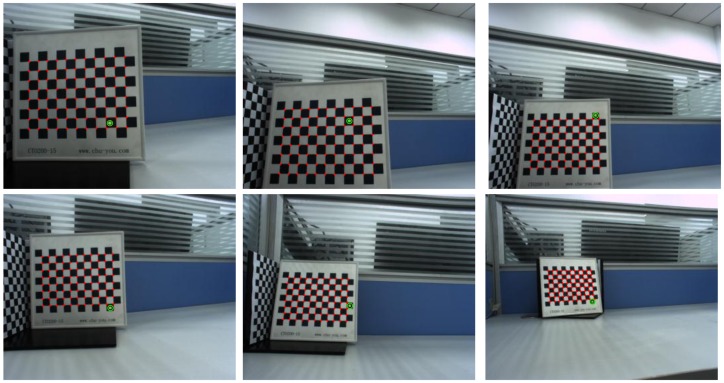
Image sample when the checkerboard is close to the edge.

Ferrara et al. [35] mentioned that the position of the checkerboard has an effect on the accuracy of calibration. We supplemented a set of calibration data of checkerboard location on the edge of the image to verify the effect of the change of checkerboard location on the accuracy of the proposed method. The data are shown in Figure 11. The calibration results are shown in Table 6. When the image is in the edge position, the calibration results are basically consistent with the internal and external parameters obtained in Table 3 and Table 4, and the re-projection errors of the internal and external parameters are slightly increased, but the difference is small. It therefore shown that the method proposed in this paper is also applicable when the collected data lie on the edge of the image.

## 6. Conclusions

In this paper, we present a convenient and fast method for calibrating a combined 1D laser ranging and monocular camera measurement system, aiming to realize an accurate measurement system fusing laser and vision. The method is easy to implement and has high calibration accuracy. The fast robust determination of the camera imaging model parameters is achieved by introducing a division distortion model. Then, a linear-plane constraint is formulated to realize robust estimation of the initial value of the laser-vision parameters. Finally, an unconstrained optimization problem is formulated using the rotation matrix parameters, and the high precision calibration of the whole measurement system is realized. The factors affecting the calibration accuracy are analyzed through simulation experiments, and the effectiveness of the proposed method is verified through real scene experiments.

## Figures and Tables

**Table 1:** The parameter statement of the system.

	Parameter	Mean
Coordinats	$O_{M}$/$O_{T}$/$O_{C}$/$O_{l}$	measurement/target/camera/laser-ranging coordinate system
	O−uv	image plane coordinate system of camera
	$T_{T2C}$	transformation matrix relating $O_{T}$ to $O_{C}$
	$R_{l2M}$, $t_{l2M}$	Extrinsic matrix of $O_{l}$ to $O_{C}$
	$R_{T2C}$, $t_{T2C}$	Extrinsic matrix of $O_{T}$ to $O_{C}$
	$\;T_{e2\hat{e}}$	transformation matrix relating the distortion center e to new distortion center $\hat{e}$
Imaging Geometry	$A_{C}$	intrinsic matrix
	$\lambda_{1}$, $\lambda_{2}$	distortion coefficients
	e	distortion center, expressed as ${\left[\begin{array}{cc} du_{0} & dv_{0} \end{array}\right]}^{T}$
	$\rho_{i}$	depth scale factor
	H	homography matrix
	$\hat{H}$	transformed homography matrix H
	$F_{H}$	fundamental matrix of distortion
	${\hat{F}}_{H}$	transformed fundamental matrix of distortion
Variable	$X_{i}^{T}$	position of the corner in the target coordinate system,
	$x_{i}^{u}$	ideal position of projection point in image plane
	$x_{i}^{d}$	actual position of projection point in image plane
	${\hat{x}}_{i}^{d}$	transformed image coordinates $x_{i}^{d}$
	$\mathbb{Q}(X^{C},Y^{C},Z^{C})$/$\mathbb{Q}(\begin{array}{cc} R_{T2M}, & t_{T2M} \end{array})$	plane equation of the target in camera coordinate system
	$\mathbb{C}(X^{C},Y^{C},Z^{C})$	linear equation of the laser beam in the camera coordinate system
	$E(A_{C},\lambda_{1},\lambda_{2},R_{T2M},t_{T2M})$	objective functions to be optimized
	$E_{dr}$	re-projection error

**Table 2:** System parameters of simulation.

Parameter	$A_{C}$	e	$\left(\lambda_{1},\lambda_{2}\right)$	$R_{l2M}$	$t_{l2M}$
Set value	$\left[\begin{array}{ccc} 850 & s & 512\\ 0 & 850 & 384\\ 0 & 0 & 1 \end{array}\right]$	$\left[\begin{array}{c} 509\\ 380 \end{array}\right]$	$\left(\begin{array}{c} 6.15\times 10^{-7}\\ 1.6\times 10^{-13} \end{array}\right)$	$\left[\begin{array}{ccc} 1 & 0 & 0\\ 0 & 1 & 0\\ 0 & 0 & 1 \end{array}\right]$	$\left[\begin{array}{c} 50\\ 0\\ 0 \end{array}\right]$
Unit	pixel	pixel	$\left(\begin{array}{c} {\mathrm{pixel}}^{-2}\\ {\mathrm{pixel}}^{-4} \end{array}\right)$	-	mm

**Table 3:** The system parameters of 1D LRF and camera.

Sensors	Parameter	Value
MER-131-210U3C (camera)	Sensor Size	${1/2}^{\prime \prime }$
	Resolution	1280 (H) × 1024 (V)
	Frame Rate	210 FPS
	Pixel Size	4.8 μm×4.8 μm
	Focuses	5 mm
	F (Relative Aperture)	1.4∼16
SKD-100 (LRF)	Wavelength	635 nm
	Range	1∼1000 mm
	Accuracy	2 mm

**Table 4:** System parameters of simulation.

Method	$A_{C}$	e	$\left(\lambda_{1},\lambda_{2}\right)$	Mean $E_{rp}$
Zhang [21]	$\left[\begin{array}{ccc} 1053.3 & 0 & 643.5\\ 0 & 1048.0 & 539.7\\ 0 & 0 & 1 \end{array}\right]\;\mathrm{pixel}$	-	$\left(\begin{array}{c} 0.1348{\;\mathrm{mm}}^{-2}\\ 0.01661{\;\mathrm{mm}}^{-4} \end{array}\right)$	0.09337 pixel
Li [30]	$\left[\begin{array}{ccc} 1059.7 & 0.1604 & 649.5\\ 0 & 1058.9 & 539.2\\ 0 & 0 & 1 \end{array}\right]\;\mathrm{pixel}$	-	$\left(\begin{array}{c} 0.1372{\;\mathrm{mm}}^{-2}\\ 0.1993{\;\mathrm{mm}}^{-4} \end{array}\right)$	0.07974 pixel
proposed	$\left[\begin{array}{ccc} 1055.2 & 0 & 647.2\\ 0 & 1054.9 & 538.1\\ 0 & 0 & 1 \end{array}\right]\;\mathrm{pixel}$	$\left[\begin{array}{c} 509\\ 380 \end{array}\right]$ pixel	$\left(\begin{array}{c} 6.15\times 10^{-7}\\ 1.6\times 10^{-13} \end{array}\begin{array}{c} {\mathrm{pixel}}^{-2}\\ {\mathrm{pixel}}^{-4} \end{array}\right)$	0.07725 pixel

**Table 5:** System extrinsic parameters and evaluation error.

Parameter	$R_{l2M}$	$t_{l2M}$	$E_{dr}$
Set value	$\left[\begin{array}{ccc} 0.9974 & 0.0052 & -0.0715\\ -0.0052 & 0.9893 & -0.1456\\ 0.0715 & 0.1456 & 1 \end{array}\right]$	$\left[\begin{array}{c} 0.5030\\ 32.6479\\ 1.859 \end{array}\right]$	0.10173
Unit	-	mm	pixel

**Table 6:** System parameters and evaluation error when checkerboard is close to edge.

Parameter	$A_{C}$	e	$\left(\lambda_{1},\lambda_{2}\right)$	Mean $E_{rp}$
Value	$\left[\begin{array}{ccc} 1057.9 & 0 & 646.9\\ 0 & 1057.2 & 536.8\\ 0 & 0 & 1 \end{array}\right]\;\mathrm{pixel}$	$\left[\begin{array}{c} 507\\ 379 \end{array}\right]$ pixel	$\left(\begin{array}{c} 6.21\times 10^{-7}\\ 1.53\times 10^{-13}\; \end{array}\begin{array}{c} {\mathrm{pixel}}^{-2}\\ {\mathrm{pixel}}^{-4} \end{array}\right)$	0.08371 pixel
**Parameter**	$R_{l2M}$	$t_{l2M}$	$E_{dr}$	
Set value	$\left[\begin{array}{ccc} 0.9962 & 0.0101 & -0.0865\\ -0.0101 & 0.9902 & -0.1381\\ 0.0855 & 0.1388 & 0.9999 \end{array}\right]$	$\left[\begin{array}{c} 0.8167\\ 31.5531\\ 1.6742 \end{array}\right]$ mm	0.1147 pixel	
